# Polygenic risk for depression and career performance

**DOI:** 10.1192/j.eurpsy.2025.348

**Published:** 2025-08-26

**Authors:** A. Hazak, J. Liuhanen, K. Kantojärvi, S. Sulkava, T. Jääskeläinen, V. Salomaa, S. Koskinen, M. Perola, T. Paunio

**Affiliations:** 1 Department of Public Health and Welfare, Finnish Institute for Health and Welfare; 2 SleepWell Research Program, University of Helsinki, Helsinki; 3 Department of Finance, Aalto University, Helsinki area, Finland; 4 Department of Economics and Finance, Tallinn University of Technology, Tallinn, Estonia; 5 Department of Psychiatry / SleepWell Research Program, University of Helsinki; 6 Department of Psychiatry; 7 Department of Psychiatry / Department of Clinical Genetics, Helsinki University Central Hospital; 8 Research Program for Clinical and Molecular Metabolism, University of Helsinki, Helsinki, Finland

## Abstract

**Introduction:**

Major depression not only impairs individual health and labour market performance but also imposes significant economic burdens on society. It is linked to substantial costs through healthcare use, lost productivity and absenteeism. Recent genome-wide association studies have identified genetic markers associated with major depression, offering new insights into its genetic risk factors. However, the potential association of these genetic risks with educational attainment and career outcomes remains underexplored. Understanding this connection is crucial for addressing the broader public health and socio-economic implications of depression risk beyond clinical populations.

**Objectives:**

This study aims to investigate the relationship between genetic risk for depression and individual career performance in the general population of Finland from 1992 to 2017.

**Methods:**

We utilised pooled data from the Finnish Finrisk (1992-2012) and FinHealth (2017) studies, which together include a population representative sample of individuals aged 25-64 (N=20,121). Genetic, survey and socio-economic registry data were integrated for this analysis. Using probit and semi-structural regression models, we examined various career performance indicators, with polygenic scores for depression (Howard *et al*. Nat Neurosci 2019; 22 343-352) as the main explanatory variable. Socio-demographic characteristics and genetic principal components were included as controls.

**Results:**

Our study revealed a negative association between higher genetic risk for depression and the likelihood of attaining higher education—an essential predictor of career success. Additionally, our study provides novel insights into how elevated polygenic risk for depression was linked to employment and self-employment rates, both directly and via educational pathways.

**Conclusions:**

These findings highlight that genetic predispositions for depression can adversely affect career prospects in the general population, suggesting that the economic burden of depression extends beyond those clinically diagnosed. As effect sizes are modest, our results imply that supportive measures and compensatory behaviours could mitigate some of the educational and career disadvantages associated with higher genetic risk for depression.

**Disclosure of Interest:**

A. Hazak: None Declared, J. Liuhanen: None Declared, K. Kantojärvi: None Declared, S. Sulkava: None Declared, T. Jääskeläinen: None Declared, V. Salomaa: None Declared, S. Koskinen: None Declared, M. Perola: None Declared, T. Paunio Consultant of: Idorsia Pharmaceuticals and Biogen (unrelated to the present work)

